# Case Report: *In situ* pulmonary artery thrombosis in a 12-year-old girl classified as systemic lupus erythematosus

**DOI:** 10.3389/fped.2024.1341188

**Published:** 2024-02-08

**Authors:** Yong Feng, Ning Chen, Bing Dai, Yunxiao Shang

**Affiliations:** Department of Pediatrics, Shengjing Hospital of China Medical University, Shenyang, China

**Keywords:** *in situ* pulmonary artery thrombosis, systemic lupus erythematosus, child, pulmonary embolism, antiphospholipid syndrome

## Abstract

*In situ* pulmonary artery thrombosis (ISPAT) is a relatively rare but potentially life-threatening complication of systemic lupus erythematosus (SLE) in children. We report the case of a 12-year-old girl who presented with fever, chest pain, and dyspnea. Immune thrombocytopenia was identified due to purpura and menorrhagia 3 months before presentation with a lowest platelet count of 12 × 10^9^/L. The sudden onset of fever, chest pain, and dyspnea were misdiagnosed as hyperinflammatory responses caused by pneumonia; these symptoms ameliorated with glucocorticoid and antibiotic treatment. The reappearance of symptoms after dose reduction of glucocorticoids and the observation of bloody bronchoalveolar lavage fluid necessitated further evaluation. Pulmonary artery thrombosis/embolism was identified using computed tomography pulmonary angiography and high D-dimer quantitative level of 4,118 μg/L (normal <252 μg/L). Ultrasonography of the deep and superficial veins of both lower limbs and renal veins revealed no thrombosis, suggesting the diagnosis of ISPAT. Further etiological evaluation revealed positive antinuclear antibodies, lupus anticoagulant, and anti-SSA antibodies, confirming SLE. Repeated normal urine analysis indicated that lupus nephritis was unlikely. Further, the negative anticardiolipin and anti-β_2_ glycoprotein antibodies and temporary positive lupus anticoagulant suggested that antiphospholipid syndrome was unlikely. The patient received anticoagulants, glucocorticoids, hydroxychloroquine, and mycophenolate therapy. Her symptoms gradually improved, and she was discharged. At the 1-month follow-up, the thrombosis had resolved. During the 1-year follow-up, her condition remained well without SLE relapse. Our experience with this case emphasizes searching for SLE in the case of ISPAT and pulmonary hemorrhages. ISPAT can occur in children with SLE and may be caused by hyperinflammatory response during SLE flare.

## 1 Introduction

Pulmonary embolism (PE) is a clinical condition caused by embolism obstruction of the pulmonary artery and its branches. PE is rare in children, with an incidence of 4.6/100,000 in all children and 57/100,000 in hospitalized children (1), significantly lower than in adults (2). During the past few decades, the incidence of PE in children has been on the rise, partially due to the generally increased awareness of the condition (3). PE can cause significant morbidity and mortality (4). It is always thought to be correlated with deep vein thrombosis (DVT), whose clot migrates to the pulmonary artery, also called classic thromboembolism PE (TE-PE). While, a static clot can build up owing to local causes and remain in the pulmonary artery, called *in situ* pulmonary artery thrombosis (ISPAT). ISPAT is often caused by endothelial dysfunction or inflammation and less often by a coagulopathy or blood stasis. It should be considered when there is no evidence of DVT (5, 6). It is reported that ISPAT occurrs at a younger age (5, 6). However, currently, little is known about the thrombophilic risk factors during the development of ISPAT in children.

**Figure 1:**
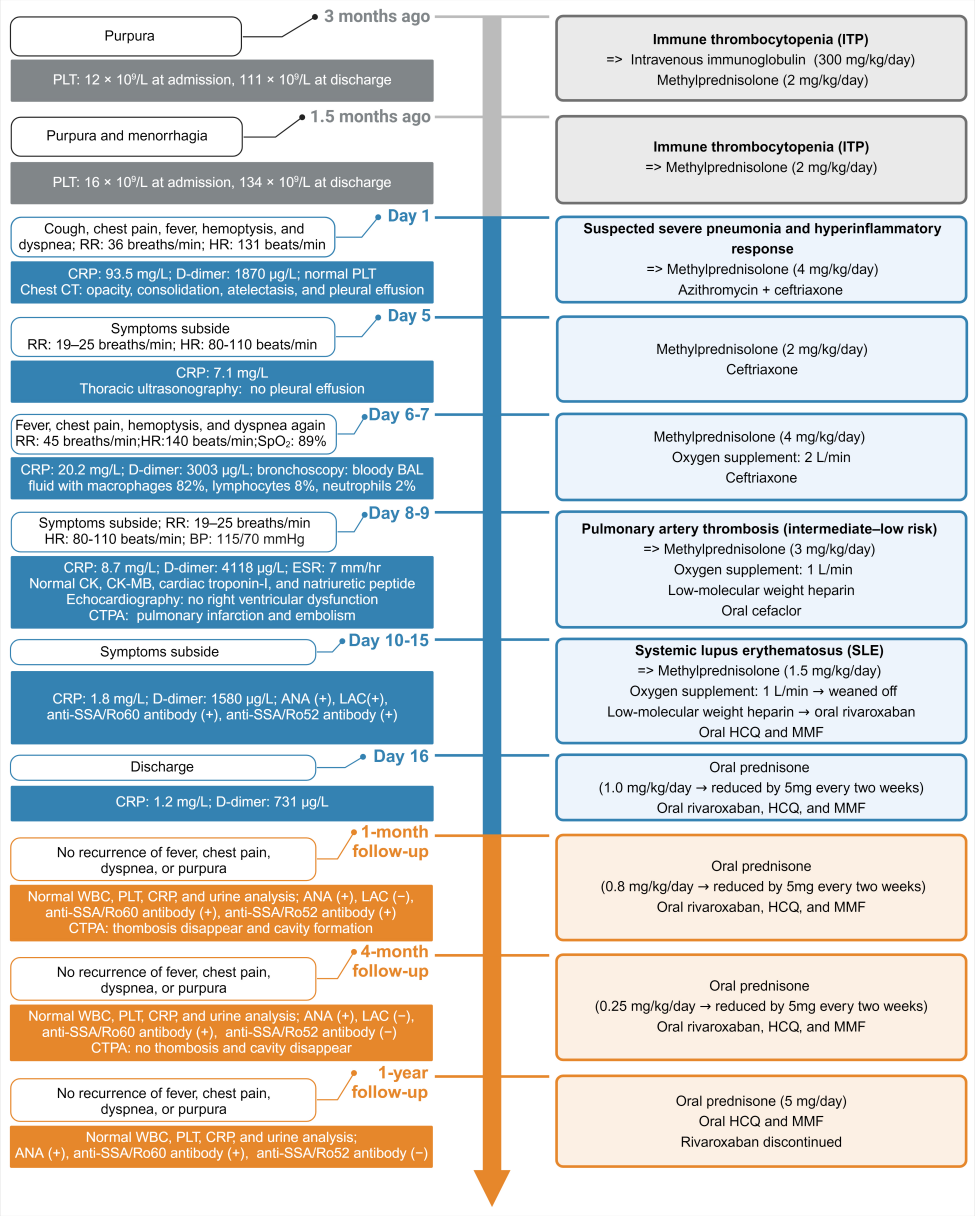
Principal clinical events and therapeutic strategies timeline. RR, respiratory rate; HR, heart rate; CRP, C-reactive protein; BAL, bronchoalveolar lavage; CK, creatine kinase; CK-MB, creatine kinase-myocardial band; CTPA, computed tomography pulmonary angiography; ANA, antinuclear antibody; LAC, lupus anticoagulant; HCQ, hydroxychloroquine; MMF, mycophenolate mofetil; WBC, white blood cell; PLT, platelet.

Systemic lupus erythematosus (SLE) is a complicated, multifactorial autoimmune disorder involving almost any organ system and is usually characterized by various clinical manifestations (7). Vascular endothelial inflammation and injury can be detected in this type of disease (8). Childhood-onset SLE can be complicated by thrombosis or embolism of different organs, and pulmonary involvement has been reported (9, 10). Among the 120 children with SLE reported by Montes de Oca et al., 11 (9%) had thrombotic episodes, and only 4 (3.3%) had TE-PE (10). TE-PE always occurs in SLE patients with antiphospholipid syndrome (APS) (11–14) or lupus nephritis (LN) (15). Herein, we report the case of a 12-year-old girl diagnosed with ISPAT caused by SLE (Figure 1).

## 2 Case presentation

A 12-year-old girl was admitted to our hospital on 23 June 2022, for 4 days of purpura. The blood test results showed a white blood cell (WBC) count of 6.16 × 10^9^/L, 85.7% neutrophils, 11.9% lymphocytes, hemoglobin level of 114 g/L, and platelet (PLT) count of 12 × 10^9^/L. The blood film showed normal platelet morphology. Testing for autoimmune disorders was performed (Table 1) and no alternative diagnosis could be identified. Immune thrombocytopenia was diagnosed and the patient received intravenous immunoglobulin at a dose of 17.5 g/day (300 mg/kg/day) for 3 consecutive days. The repeat PLT count revealed 35 × 10^9^/L, indicating poor response to intravenous immunoglobulin. The bone marrow analysis was performed and demonstrated normal trilineage hematopoiesis. Then, the patient received 4 days of methylprednisolone therapy at a dose of 120 mg/day (2 mg/kg/day). Subsequently, her PLT count increased to 111 × 10^9^/L. She was discharged, and oral prednisone was continued at a dosage of 60 mg/day for a week without tapering. About 1 month later, the patient was readmitted for 3 days of menorrhagia and a day of purpura. Her PLT count decreased to 16 × 10^9^/L. Methylprednisolone was administered at a dosage of 120 mg/day for 1 week. The PLT count increased to 134 × 10^9^/L. After 8 days of hospitalization, she was discharged.

**Table 1:** Laboratory characteristics of the patient.

Items	24 June 2022	7 August 2022	22 September 2022	9 November 2022	8 March 2023	29 August 2023	Reference range
ANA	≥1:640	≥1:640	1:5,120	1:2,560	1:1,280	1:1,280	<1:80
Anti-SSA/Ro60	>8.0	4.0	6.3	10.7	5.5	>8.0	<0.8
Anti-SSA/Ro52	(−)	2.3	1.7	1.3	(−)	(−)	<0.8
Anti-dsDNA	(−)	(−)	(−)	(−)	(−)	(−)	<0.8
LAC	(−)	(−)	(+)	(−)	(−)	UK	(−)
aCL (IgG, IgM, and IgA)	UK	(−)	(−)	(−)	(−)	UK	<20
aβ_2_GPI (IgG, IgM, and IgA)	UK	UK	(−)	(−)	UK	UK	<20
ANCA	UK	UK	(−)	(−)	UK	UK	(−)
Complement C3 (g/L)	1.162	1.197	0.984	1.0	1.02	1.17	0.74–1.4
Complement C4 (g/L)	0.16	0.159	0.138	0.164	0.184	0.2331	0.12–0.36
ESR (mm/h)	UK	UK	7	8	UK	14	0–20

ANA, antinuclear antibody; LAC, lupus anticoagulant; aCL, anticardiolipin antibody; aβ_2_GPI, anti-β_2_-glycoprotein I antibody; anti-dsDNA, anti-double-stranded DNA; ESR, erythrocyte sedimentation rate; UK, unknown; (−), negative; (+), positive.

**Figure 2:**
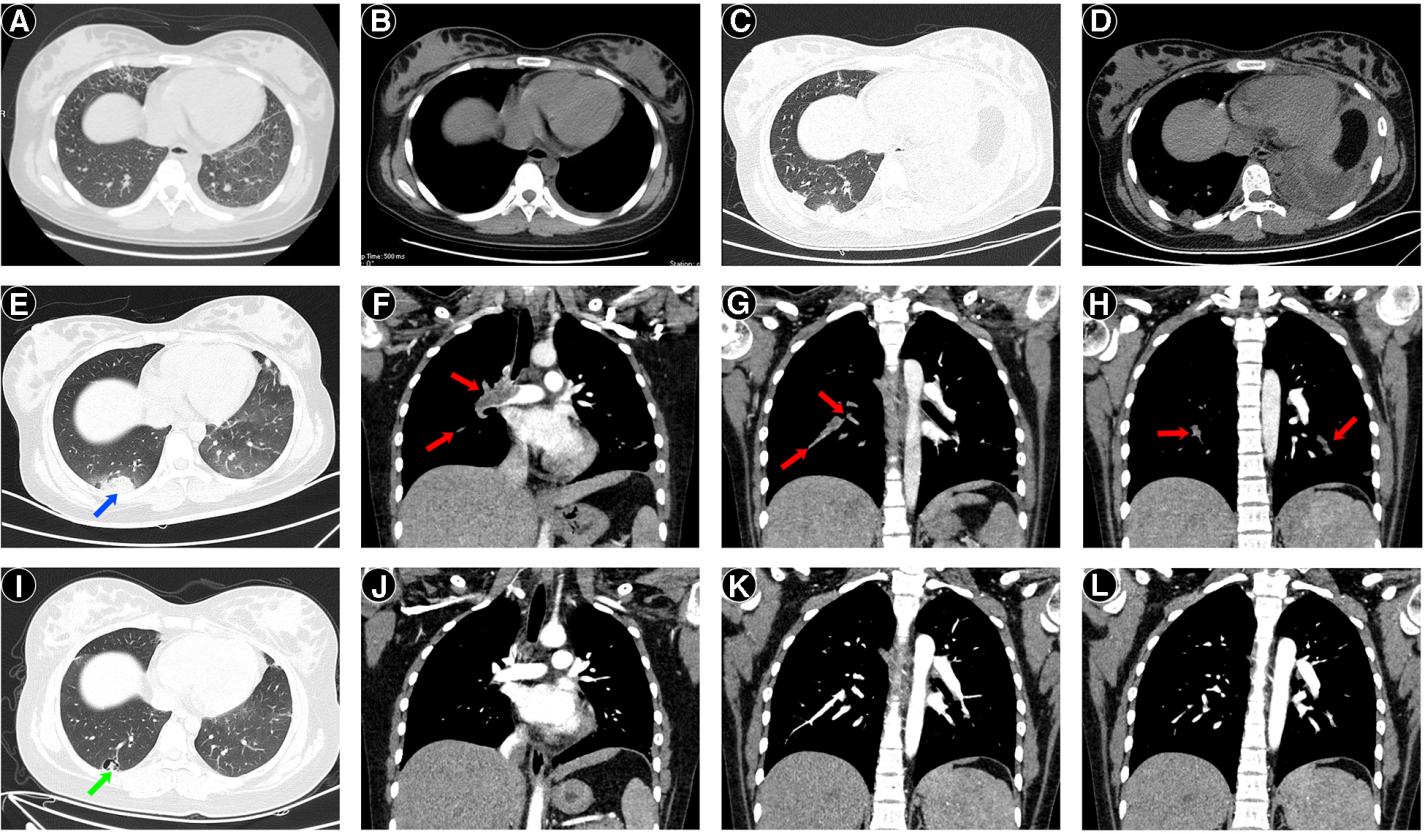
Chest computed tomography (CT) images of the patient. Two days before admission, CT revealed a few infiltrates in the right middle lobe and left lower lobe, with a small amount of left pleural effusion (**A,B**). On day 2 after admission, CT revealed opacity in the right lower lobe's dorsal segment, consolidation, atelectasis of the left lower lobe, and left pleural effusion (**C,D**). On day 7 after admission, computed tomography pulmonary angiography (CTPA) at the lung windows (**E**) showed peripheral wedge-shaped density on the right side (blue arrow), representing pulmonary infarction. Sagittal reconstruction of CTPA showed filling defects (red arrows) in the right main pulmonary artery (**F**) and pulmonary branches (**G**), and the left inferior pulmonary artery (**H**), consistent with clots. One month after discharge, follow-up CTPA showed the formation of a cavity (green arrow) (**I**) and the disappearance of filling defects (**J–L**).

On 14 September 2022, she was admitted again for a month-long cough, 2 days of chest pain and tightness, and a day of fever. The patient first visited a local hospital and was treated with cephalosporins for 10 days. Two days before admission, the patient developed chest tightness, chest pain, and dyspnea. The chest pain was localized to the right chest wall and back, and was worsened by breathing. One day before admission, she got fever (maximum: 39.3°C, twice daily), and her cough worsened. She visited another hospital, and the blood test results showed a WBC count of 7.4 × 10^9^/L, 83.2% neutrophils, 8.9% lymphocytes, hemoglobin level of 98 g/L, PLT count of 146 × 10^9^/L, an elevated C-reactive protein (CRP) level (66.8 mg/L, normal <6 mg/L), normal serum cardiac enzymes, and normal arterial blood gas analysis. Chest computed tomography (CT) revealed infiltrates in the right middle and left lower lobes, with a small amount of left pleural effusion (Figures 2A,B). The patient was treated with cefodizime with no improvement.

On admission, physical examination revealed a body temperature of 39.2°C, blood pressure of 117/61 mmHg, heart rate (HR) of 131 beats/min, respiratory rate (RR) of 36 breaths/min, and oxygen saturation of 95% (room air). Chest auscultation revealed no rales and normal vocal or tactile fremitus. Heart sounds were normal, with no murmur. The remaining physical examination findings were unremarkable. The blood tests showed a WBC count of 7.3 × 10^9^/L, hemoglobin level of 98 g/L, PLT count of 110 × 10^9^/L, elevated CRP level of 93.5 mg/L, and procalcitonin level of 0.1 ng/mL (normal <0.05 ng/mL). Arterial blood gas analysis revealed a pH of 7.50, PaCO_2_ of 27.0 mmHg, and PaO_2_ of 87.1 mmHg (room air). Her liver and kidney functions, lactate dehydrogenase, and cardiac enzyme levels were normal. Coagulation testing showed a prothrombin time of 12.8 s (normal range 9.4–12.5 s), activated partial thromboplastin time of 17 s (normal range 21–37 s), international normalized ratio of 1.2 (normal range 0.8–1.2), D-dimer quantitative level of 1,870 μg/L (normal <252 μg/L), and normal fibrinogen level. The urine analysis results were normal without proteinuria. Serum antibody tests for *Mycoplasma pneumoniae* (MP) IgM and *Chlamydia pneumoniae* IgM and polymerase chain reaction (PCR) tests of nasopharyngeal swabs for MP, adenovirus, respiratory syncytial virus, and influenza virus were negative. Echocardiography revealed a small pericardial effusion with a normal ejection fraction (62%). She was treated with azithromycin and ceftriaxone as antibiotic therapy.

On day 2, the chest pain, fever, and cough continued deteriorating. Small streaks of blood were observed in the mucus. Chest auscultation revealed diminished breathing sounds on the left side. Repeat chest CT showed an opacity on the right lower lobe's dorsal side, consolidation and atelectasis of the left lower lobe, and moderate left pleural effusion (Figures 2C,D). Thoracic ultrasonography revealed left pleural effusion (depth: 4.3 cm). The rapid deterioration of chest pain, fever, and cough, as well as the rapid progression of CRP, lung consolidation, and pleural effusion, suggested a hyperinflammatory response. Thus, methylprednisolone therapy at 120 mg twice daily (4 mg/kg/day) was administered for 3 consecutive days. The fever, chest pain, and dyspnea resolved. On day 5, the CRP decreased to 7.1 mg/L, and repeat thoracic ultrasonography revealed no pleural effusion. The dose of methylprednisolone was reduced to 60 mg twice daily (2 mg/kg/day).

On day 6, the patient became febrile again (maximum: 38°C). She suddenly experienced dyspnea (RR, 45 breaths/min), tachycardia (HR, 140 beats/min), hypoxemia (oxygen saturation, 89%), and right subscapular pain, which worsened after coughing. Moist rales were heard on auscultation. Oxygen was administered at 2 L/min to maintain normal oxygen saturation. Considering the uncontrolled hyperinflammatory response, the methylprednisolone dose was increased to 120 mg twice daily (4 mg/kg/day). The chest pain and dyspnea improved. On day 7, a bronchoscopy was performed, and bronchoalveolar lavage (BAL) fluid was collected. BAL fluid was bloody, particularly when suctioned from the right lung. However, chest CT on day 2 revealed that the lesions mainly involved the left lung. BAL fluid PCR assays for MP, adenovirus, respiratory syncytial virus, influenza virus, and *Mycobacterium tuberculosis* and the cultures for respiratory bacteria were negative. The level of cytokine interleukin-6 in BAL fluid was elevated (3,220.38 pg/mL, normal ≤5.4 pg/mL). The differential nucleated cell counts of BAL fluid were normal. Thus, the bloody BAL fluid could not be due to pneumonia, and PE was suspected. Emergency computed tomography pulmonary angiography (CTPA) revealed peripheral wedge-shaped densities in the lower lobes of both lungs, representing pulmonary infarctions and filling defects in the right main pulmonary artery, left inferior pulmonary artery, and their branches, which were consistent with the presence of clots (Figures 2E–H).

The risk of early death was evaluated, which revealed normal blood pressure, simplified Pulmonary Embolism Severity Index of 1 point (oxygen saturation <90%), normal right ventricle function, and normal cardiac troponin level. The patient had an intermediate–low early mortality risk according to the guidelines (16). The anticoagulation was initiated with subcutaneous low-molecular-weight heparin at a dose of 100 IU/kg twice daily for 7 consecutive days. Methylprednisolone was reduced to 80 mg twice daily (3 mg/kg/day), and the antimicrobial therapy was changed to oral cefaclor. The etiology of thrombosis was further evaluated. Ultrasonography of the deep and superficial veins of both lower limbs and renal veins revealed no thrombosis. The antinuclear antibody (ANA) titer was 1:5,120 (nuclear granular-type). Positive lupus anticoagulant (LAC), anti-SSA/Ro60, and anti-SSA/Ro52 antibody results were noted. Anticardiolipin antibody (aCL) and anti-β_2_-glycoprotein I antibody (aβ_2_GPI) IgG, IgM, and IgA, and anti-double-stranded DNA (anti-dsDNA) antibodies were negative. Serum complement levels (C3 and C4), immunoglobulins (A, G, and M), rheumatoid factor, serum ferritin, and erythrocyte sedimentation rate (ESR) were normal. Subsequently, childhood-onset SLE was diagnosed based on an ANA titer of 1:5,120, fever, pleural and pericardial effusion, thrombocytopenia, and positive LAC (7, 17). The results of repeated urine analysis checking for proteinuria were normal. Hydroxychloroquine (HCQ) and mycophenolate mofetil (MMF) were administered at 100 mg and 600 mg/m^2^ twice daily, respectively.

On day 11, the patient's condition improved without fever or chest pain. The oxygen saturation was 98% on 1 L/min of oxygen. The methylprednisolone dose was reduced to 80 mg/day (1.5 mg/kg/day). On day 14, the patient was weaned off supplementary oxygen. Anticoagulation therapy was changed to rivaroxaban administered orally at a dose of 15 mg twice daily. On day 16, the patient was discharged, and oral rivaroxaban, prednisone, HCQ, and MMF were continued. Rivaroxaban was administered at 15 mg twice daily for 21 days, followed by 20 mg/day for maintenance. Oral prednisone was initiated at a dose of 60 mg/day and was reduced by 5 mg every 2 weeks.

She was regularly followed-up and remained well, with no recurrence of fever, chest pain, dyspnea, or purpura. Repeated CTPA showed resolution of consolidations, thrombosis, pleural effusion, and cavity formation in the dorsal side of the right lower lobe at the 1-month follow-up (Figures 2J–L). At the 4-month follow-up, CTPA revealed no recurrence of thrombosis and disappearance of the cavity, which progressed to a fibrotic scar. Renal and cardiac ultrasound were performed at the 1- and 4-month follow-ups and revealed to be normal. The laboratory characteristics during follow-up are summarized in Table 1. The ANA titer and anti-SSA/Ro60 antibody remained positive, and the anti-dsDNA, aCL, and aβ_2_GPI antibodies remained negative. LAC and anti-SSA/Ro52 antibody turned to negative at the 1- and 4-month follow-ups, respectively. The WBC and PLT counts, CRP, complement C3 and C4 levels, and urine analysis remained normal. HCQ and MMF were then administered. At the 1-year follow-up, the dose of oral prednisone was gradually reduced to 5 mg/day and rivaroxaban was discontinued owing to no recurrence of thrombosis.

## 3 Discussion

In this case, the thrombocytopenia was presumed to be autoimmune thrombocytopenic purpura. The poor response to intravenous immunoglobulins and good response to glucocorticoids raise the risk of a different underlying diagnosis, particularly SLE. The thrombocytopenia of this patient was severe with lowest PLT count of 12 × 10^9^/L, which had been reported in SLE. In a large single-center cohort study, 28% of SLE patients had a PLT count of less than 20 × 10^9^/L (18). SLE is a complex autoimmune disorder in which pathogenic autoantibodies and immune complexes damage the tissues and cells. The diagnosis of SLE in this patient meets the classification criteria recommended by the European League Against Rheumatism and the American College of Rheumatology (EULAR/ACR-2019) (7). First, the ANA >1:80 met the entry criterion. According to the weighted criteria, the patient got a total score of 13 points. In the clinical domain, the weight for fever, thrombocytopenia, and pleural or pericardial effusion were two points, four points, and five points, respectively. In the immunology domain, the weight for positive LAC was two points. EULAR/ACR-2019 recommended that a patient can be classified as SLE if the total score ≥10 points with at least one clinical criterion. A study demonstrated that the EULAR/ACR-2019 total score ≥13 points, against the initially proposed ≥10 points, was most appropriate to classify childhood-onset SLE (19). This patient was classified as SLE according to the EULAR/ACR-2019 classification criteria, which has a specificity of 93% and sensitivity of 96% (7). Disease classification criteria have many imperfections, and have been developed mainly to homogenize groups of patients for research purposes, not for diagnostic purposes. The patient in our case study clearly showed a predominant vascular phenotype, severe thrombocytopenia, and positive LAC, with no specific SLE or laboratory manifestations. The negative anti-dsDNA and anti-Sm antibodies, and normal ESR, C3, and C4 levels during flare, make the diagnosis of SLE doubtful. Studies suggested that anti-dsDNA and anti-Sm antibodies had high specificity and low sensitivity for the diagnosis of SLE, and are reported to be associated with renal involvement, which was not seen in our case (20). The ESR and complements were not performed routinely at presentation, but after methylprednisolone therapy for a week. In a recent study of patients with probable lupus, only 36% had low C3 and/or C4 historically even though 30% transitioned to SLE at follow-up (21). CRP increases significantly in SLE patients with concomitant infection, but rarely during lupus flare without infection (22). It has been found that the ESR/CRP ratio above 15 was significantly correlated with SLE flare (23). The elevated CRP in this patient might be due to pleural/pericardial lesion or potential macrophage activation (24).

Recently, childhood-onset SLE has been linked to single gene mutations, defining the concept of monogenic lupus. Genes associated with monogenic lupus can be grouped in at least three functional categories, including complement deficiencies, type-I interferonopathies, and adaptive immunity tolerance breakdown (25). Typically, monogenic lupus is presenting early in life, usually at <5 years of age, with severe disease manifestations (26). Our case is a 12-year-old girl without familial SLE or involvement of the skin, kidney, and nervous systems. She had good response to routine treatment for SLE. Thus, monogenic lupus seems to be unlikely based on the current evidence. However, the manifestations of monogenic lupus are very heterogeneous, depending on the affected gene. Monogenic lupus cannot be completely ruled out without genetic testing, which was not performed because of the lack of parental consent. A long-term follow-up is needed to see whether the patient will develop typical SLE in the future.

Venous thromboembolism occurs when more than one component of Virchow's triad is activated: blood stasis, hypercoagulability, and endothelial injury (27). TE-PE, often caused by blood stasis and hypercoagulability, is associated with DVT (16). Blood clots originate from the deep venous system of the lower extremities and, less frequently, from the pelvic, renal, or upper extremity veins. The clot is dislodged to form an embolus, which subsequently migrates and lodges into a major or smaller branch of the pulmonary circulation, leading to TE-PE. However, no DVT was observed in the present case upon ultrasonographic evaluation of the lower extremities and renal veins. In a study on the origin of PE, up to 50% of adult patients had no obvious source of emboli (28). This phenomenon was also observed in children (6). This suggests that PE may be a local phenomenon that starts in the pulmonary arteries, also called ISPAT. Unlike TE-PE, ISPAT is often caused by endothelial injury and platelet activation due to hypoxia, proinflammatory cytokines, or genetic mutations (29). ISPAT has been reported in chronic obstructive pulmonary disease, severe infections, asthma, tumors, sickle cell anemia, surgery, viral pneumonia (influenza and COVID-19), autoimmune diseases such as SLE, and other conditions (29, 30).

Childhood-onset SLE can be complicated by ISPAT due to pulmonary artery endothelial injury. In this case, endothelial damage to the pulmonary vessels caused by pathogenic autoantibodies and proinflammatory cytokines might be the underlying mechanism of ISPAT. The transient presence of LAC did not support APS (31) but was a main predictor of thrombosis in SLE (32). In this case, the symptoms and elevated CRP levels were relieved after methylprednisolone therapy and recurred after dose reduction. Elevated IL-6 levels were also found in BAL with normal cell counts, which might not be due to infection. Elevated CRP and IL-6 levels indicate a hyperinflammatory response caused by an SLE flare, further exacerbating the prothrombotic state. The endothelial damage as the causative factor of ISPAT can be given as acquired as suggested by other models of diseases such as Behçet's syndrome (33), the Hughes–Stovin syndrome (34), and Buerger's vasculitis (35). Behçet's syndrome is a multisystem vasculitis and often complicated solely with ISPAT but not TE-PE (33). Emad et al. reported that 44 (77.2%) patients with the Hughes–Stovin syndrome, a systemic disease characterized by pulmonary vasculitis, had *in situ* thrombosis in pulmonary artery aneurysms (34). Therefore, ISPAT could occur in a patient with SLE without APS or LN, which has rarely been reported.

APS, a systemic autoimmune disorder, typically presents with arterial and venous thrombosis and thrombotic microangiopathy (36). The positive LAC during thrombosis met the entry criteria for APS classification (31). However, LAC tests done 3 months before and 4 months after the positive LAC were negative. There may be doubt about the negative LAC results with anticoagulation during follow-up, resulting in potentially false-positive or false-negative results. According to the guideline, LAC detection should be deferred until anticoagulation is discontinued (37). However, studies demonstrated that rivaroxaban prolonged RVVT screen/confirm ratio, leading to false-positive identification of LAC (38, 39). Thus, the use of rivaroxaban did not influence the LAC tests in this case. Nevertheless, the influence of anticoagulation should be considered in LAC detection and interpretation. The antibodies aCL and aβ_2_GPI remained negative during repeat tests. The antiphosphatidylserine-prothrombin antibodies testing may have diagnostic relevance for APS, which was not available in our hospital (40). Therefore, the single positive LAC did not support the diagnosis of APS in the present case (31).

Pulmonary thrombosis can lead to pulmonary hemorrhage, which is always alveolar hemorrhage, caused by extravasation of erythrocytes from the pulmonary capillaries. Pulmonary hemorrhage is commonly seen in the occlusion of the distal pulmonary arteries, but not the central artery (41). Therefore, the patient had hemoptysis and bloody BAL fluid. On chest CT, the most common finding of pulmonary hemorrhage is ground-glass or airspace opacities, and lung apices and costophrenic angles are rarely affected (42). Findings on chest CT may be negative in up to 50% of the cases (43). In the present case, no specific treatment was given for hemorrhage. Tranexamic acid (TA), an antifibrinolytic agent, may be effective in reducing the amount of hemoptysis and controlling the bleeding, especially by the aerosol administered (44). Unfortunately, nebulized TA has not been approved by the China National Medical Products Administration for use in children, so it cannot be tried in this case. Nebulized TA in pulmonary hemorrhage in children was described in case reports, which still needs further evaluation. After the acute phase, oral anticoagulants were continued for preventing recurrence of thrombosis over the long-term. Studies indicated that the use of rivaroxaban in high-risk patients with APS was associated with an increased risk of recurrent thrombosis compared with warfarin (45). Rivaroxaban was administrated in our case due to the initial diagnosis of SLE instead of APS and the convenience of rivaroxaban without routine coagulation monitoring. Fortunately, the repeat CTPA, renal ultrasound, and cardiac ultrasound revealed no thrombosis recurrence. However, warfarin seems to be more suitable than rivaroxaban in this case due to the suspected APS with previous arterial events and positive LAC.

In conclusion, ISPAT should be suspected when chest pain, hypoxemia, and hemoptysis cannot be completely attributed to pneumonia or other common diseases, particularly in patients with high plasma D-dimer levels. The study emphasizes the need for searching autoimmune disorders in the case of ISPAT and pulmonary hemorrhages. ISPAT can occur in patients with childhood-onset SLE without APS or LN, and may be caused by hyperinflammatory response during SLE flare.

## Data Availability

The original contributions presented in the study are included in the article/Supplementary Material, further inquiries can be directed to the corresponding author.
